# Field efficacy of ethnomedicinal plant smoke repellency against *Anopheles arabiensis* and *Aedes aegypti*

**DOI:** 10.1016/j.heliyon.2021.e07373

**Published:** 2021-06-24

**Authors:** Abenezer Wendimu, Wondimagegnehu Tekalign

**Affiliations:** Department of Biology, College of Natural and Computational Sciences, Wolaita Sodo University, PO Box 138, Wolaita Sodo, Ethiopia

**Keywords:** Repellent plants, Mosquito bites, Smoke repellency, Plant based products, Malaria, Dengue

## Abstract

The repellency effect of smoke from burning *Azadirachta indica, Eucalyptus camaldulensis* and *Ocimum forskolin* plants to reduce human-mosquito biting activity. Ground mixed powders of the plant leaves produced smoke by direct burning and thermal expulsion on the traditional stoves in experimental huts against *An. arabiensis* and *Ae. aegypti.* A four-by-four Latin-square design was used to assign treatment and control experimental huts over different nights. In the treatment huts, the percent repellency of the smoke produced by burning powdered plant mixtures of the plants were determined by reduction mosquito density. There was a reduction on *An. arabiensis* (93.75%, P < 0.001) and *Ae. aegypti* (92%, P < 0.001) respectively, for huts with burning powder versus no treatment. Overall, plant mixed powders tested by both methods of application offered significant protection (>90%) against both mosquito species tested and has the potential to be used as an alternative mosquito control method.

## 1 Introduction

Mosquitoes are vectors of pathogens of medical importance that affect 70 billion people every year around the world (WHO, 2017). Globally 229 million new malaria infections and 409, 000 deaths were recorded in 2019 (WHO, 2020). Each year, more than 400, 000 people die of malaria. An estimated two thirds of deaths are among children under the age of five (WHO, 2020).

Malaria vaccines are not available for protection of malaria as well as any mosquito-born viruses except yellow fever and Japanese encephalitis. Therefore, protection from mosquito bites is one of the best strategies to prevent mosquito-born diseases or reduce their incidence.

Repellents extracted from plants are effective in protecting against mosquitoes and mosquito-transmitted diseases (Maia and Moore, 2011). Burning of fresh and dried leaves of plants belonging to the family Lamiaceae, Poaceae and Pinaceae are a widely used mosquito protection measure in the rural areas of Ethiopia and other tropical areas (Seyoum et al., 2002a,b, Karunamoorthi et al., 2009a,b, Dugassa et al., 2009). Smoke from plants to repel *Anopheles* mosquito species was 90.1% effective by *Ostostegia integrifolia* (Karunamoorthi et al., 2009a & b), 79.8% effective by *Olea* *europaea*, and 44.5% by *Ocimum suave* (Seyoum et al., 2003; Dugassa et al., 2009). Hanging the fresh leaves of *Ocimum* *canum* in the home was effectively provided 63.6% protection from mosquito bites in Guinea Bissau (Moore et al., 2007). The live potted plants of *Oc*. *americanum*, *Oc*. *kilimandscharicum* and *Oc*. *suave* was reported to have repellent properties providing on average of 39.7%, 44.45%, and 44.45% protection from mosquito bites in western Kenya, respectively (Seyoum et al., 2002a; b, 2003).

Malaria is a serious public concern in Ethiopia, 75% of the land and 60% of the population are exposed to the disease. The disease has been consistently reported as one of the top three leading causes of outpatient visits, admissions, and deaths among all age group in Ethiopia (Berhe et al., 2019). There are about 5–6 million annual confirmed malaria cases in Ethiopia (FMOH, 2012). Dengue fever is a viral disease primarily transmitted by *Aedes aegypti* mosquitoes. Outbreaks in eastern and central Ethiopia were reported during 2014–2017 (Degife et al., 2019; Gutu et al., 2021). During the entomological assessment from urban and rural households in Offa district, Wolaita zone almost 70 km from the study area, the presence of *Aedes* mosquitoes were witnessed (Tufa et al., 2020).

Plant-based products are believed to be safer than synthetic insecticides (Regnault-Roger et al., 2012; Wendimu and Tekalign, 2020). However, plant based products have so far received little attention in Ethiopia. This study was carried out in this regard to evaluate the field efficacy of some ethinomedicinal plants smoke repellency against *An. arabiensis* and *Ae. aegypti* by direct burning and thermal expulsion application methods in the Diguna Fango district, Wolaita, Ethiopia.

## 2 Materials and methods

### 2.1 Study area

**Figure 1:**
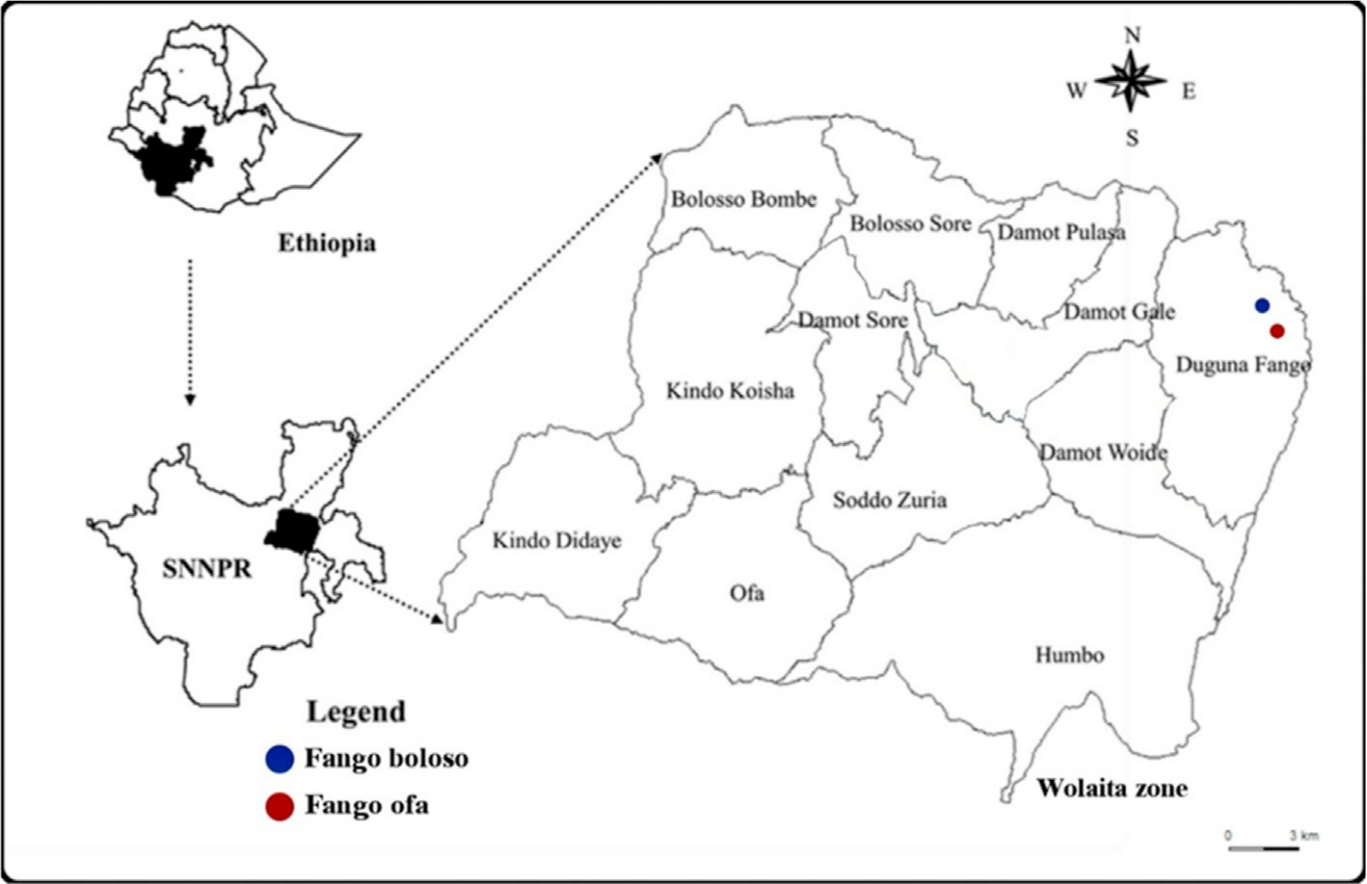
Map showing the location of the study area at Diguna Fango, Ethiopia. *Note*: SNNPR = Southern Nations Nationalities and Peoples' Region. Blue color = Fango Boloso kebele, Red color = Fango Ofa kebele.

The study was conducted in small villages in Diguna Fango district located at 6^o^57′57.1″N 38^o^02′15.7″E in the Southern Region, Wolaita zone, Ethiopia (Figure 1). The area is located 395 km south of the capital Addis Ababa, and 63km east of Wolaita Sodo town, where the Bilate River crosses the main road to Sidama Region of 1495 m above sea level (m.a.s.l). The Bilate River during the rainy season floods in low lying areas creating swampy or temporary large pools. The mean annual rainfall and temperature of the area is about 700 mm and 21 °C, respectively.

### 2.2 Test plants

Three candidate plant species Neem (*Azadirachta indica),* Red gum (*Eucalyptus camaldulensis)* and Basil (*Ocimum forskolei*) were selected for field efficacy studies (Kebede et al., 2010; Seyoum et al., 2002a, b, and 2003).

### 2.3 Preparation of plants for smoke toxicity test

The leaves of the plants were collected from road side and around local farmlands of Bilate tena (Dimtu) town following the collection techniques provided by British Columbia Ministry of Forests (1996). The collected plant leaves were dried under room temperature and was then powdered individually using traditional coffee grinding mill (‘Mekocha’ in Amharic language, which is the national official language of Ethiopia). Equal amount of the powdered leaves then thoroughly mixed and made available for experimentation.

### 2.4 Experimental houses selection

Repellency of smoke from the mixture of plant powders was tested against mosquitoes at eight traditional village houses (huts) were selected from two nearby kebeles (small units of the district); such as Fango Boloso and Fango Ofa. Four huts were used to determine the repellency of smoke from plant mixtures by direct burning and the other four were used to determine the efficacy of application of plants by thermal expulsion or thermal expelling: the act of driving out. Thermal explusion is the way to put the dried plants leave powder up on a thin hot metal plate which is placed above the lightened charcoal in a traditional stove and letting it to smoke slowly. The experimental huts are made of wood and the roofs are covered with grass (this type of hut is called ‘Sar-bet’ in the Amharic language). The walls are covered with mud in which open eaves, unscreened small holes, and doors allow mosquitoes to get in and out of the house freely.

### 2.5 Repellency tests

The repellent effect of the smoke from the leave of selected plants Neem (*Azadirachta indica*), Red gum (*Eucalyptus camaldulensis*) and Basil (*Ocimum forskolei*) leave powder were evaluated individually against *Ae*. *aegypti* and *An. arabiensis* by direct burning and thermal expulsion methods of application under normal field conditions. A four-by-four Latin-square design was used for both experiments in four treatment huts. The treatment and control huts were randomly assigned by rotation in consecutive treatment nights. The tests were carried out with an intervening period of one night to avoid the potential residual effect of the plants by periodic thermal expulsion and direct smoking. The tests were replicated for four treatment nights of the weeks. The four treatment nights considered in the study were called N_1_ (night one), N_2_ (night two), N_3_ (night three) and N_4_ (night four). In each treatment night of the weeks, there were four treatment huts with there corresponding control huts. Field evaluation repellency of smoke resulting from burning plants powders was performed with eight local volunteers (males, 15–25 years of age). The experiments were carried out in two independent blocks. Block one was for direct burning and block two was for thermal explusion. With in each blocks there were two groups, experimental and control. The first four volunteers were assigned for experimental group and the rest four were for control group. The treatment huts in the W_1_ (week one), was fumigated by the smoke from 7:00 p.m. to 11:00 p.m while adding 50g of the powder in 40 min interval directly on the traditional stove (for direct burning). The treatment huts in all the rest weeks (W_2_–W_4_) were fumigated by the smoke from 6:00 p.m. to 10:00 p.m while adding 50g of the powder in 40 min intervals in both direct burning and thermal explusion methods of application. The volunteers in the treatment and control huts collected all mosquitoes as they landed on their pant legs rolled up and exposed forearm (lower half of the arm) for a 20 min period was followed by a 5 min break before the next collection was conducted. Each day the volunteers were rotated between huts and the treatments remained fixed using a Latin Square design.

Thermal explusion was carried out by adding plant powders on top of thin hot metal plate which was then placed above the burning charcoal in the traditional stove as described by Seyoum et al. (2002a. 2003). Collectors began collecting mosquitoes within 20 min of starting the treatment followed by 5 min break from 6:00 p.m - 1:00 p.m the entire collection time was 40 min without the break. From 1:00 p.m - 2:00 p.m also the entire collection time was only 40 min without the break. Each hour holds two collection rounds. The first round is 20 min and the second round is also 20 min, totally 40 min. The remaining 10 min in the middle were used for preparation after break before the next collection began.

### 2.6 Species identification

Adults mosquitoes were morphologically identified to species using the dichotomous key to Gillies and Coetzee (1987) and Service (2012). No DNA was extracted from the mosquitoes for PCR analysis.

### 2.7 Data analysis

The repellent effect of the smoke from mixed powder of the plants were analyzed for differences between experimental units by generalized linear modeling (ANOVA) of the relationship between mosquito collections in the control (C) and in treatment huts (T). The repellence index (R) was estimated as percent, where % R = (C-T)/C × 100%, where C and T were the mean number of mosquitoes collected in the control and the treatment huts, respectively (Yap et al., 1998; Sharma and Ansari, 1994). Tukey's test was conducted to compare responses to the smoke repellency in the landing assays by using Minitab® 18 statistical package for Windows, version 10.

### 2.8 Ethical considerations

Although these studies present a minimal risk to participants, has been conducted in accordance with the declaration of Helsinki that provides guidance for the researcher to protect research subjects. The study was approved by the Institutional Research Review Board (IRB) of Wolaita Sodo University.

## 3 Results

The repellency of smoke by direct burning and thermal explusion against two biting mosquito species (*Ae. aegypti* and *An. arabiensis*) over the treatment nights are shown in the Tables 1 and 2, respectively. The comparative repelling efficacy of smoke against two biting mosquito species (*Ae. aegypti* and *An. arabiensis*) over the treatment nights by direct burning and thermal explusion is shown in Tables 3 and 4, respectively.

**Table 1:** The repellent effect of smoke by two methods for four treatment nights in individual huts by direct burning.

Weeks	Condition	Species	Total number of mosquitos collected at human bait over four treatment nights				Mean ± SE
			1	2	3	4	
W_1_	T	*An. arabiensis*	3	1	5	2	2.75 ± 0.85
		*Ae. aegypti*	0	0	1	0	0.25 ± 0.25
	C	*An. arabiensis*	45	28	47	30	37.5 ± 4.94
		*Ae. aegypti*	10	8	12	7	9.25 ± 1.10
W_2_	T	*An. arabiensis*	1	4	1	5	2.75 ± 1.03
		*Ae. aegypti*	1	0	0	2	0.75 ± 0.47
	C	*An. arabiensis*	43	29	36	31	34.75 ± 3.11
		*Ae. aegypti*	9	6	3	0	4.5 ± 1.93
W_3_	T	*An. arabiensis*	2	1	3	3	2.25 ± 0.47
		*Ae. aegypti*	1	0	1	0	0.50 ± 0.28
	C	*An. arabiensis*	41	23	32	40	34.0 ± 4.18
		*Ae. aegypti*	6	5	8	9	7.00 ± 0.91
W_4_	T	*An. arabiensis*	1	3	1	1	1.50 ± 0.50
		*Ae. aegypti*	0	0	0	1	0.25 ± 0.25
	C	*An. arabiensis*	29	32	25	39	31.25 ± 2.95
		*Ae. aegypti*	1	9	6	5	5.25 ± 1.65

SE: Standard error. T: Treatment. C: Control. W: Week.

**Table 2:** The mean repellent index (R) of mosquitoes collected at human bait in control and treatment huts by direct burning.

The mosquito species	Conditions	Mean number collected	The mean repellent index (R)	P-Value∗
*An. arabiensis*	Treatment	9.25	93.27	P = 0.0021
	Control	137.5		
*Ae. aegypti*	Treatment	1.75	93.26	P = 0.0003
	Control	26		

∗P**-**Value obtained from Tukey's test at α = 0.05.

**Table 3:** The repellent effect of smoke by two methods for four treatment nights in individual huts by thermal exclusion.

Weeks	Condition	Species	Total number of mosquitos collected at human bait over four treatment nights				Mean ± SE
			1	2	3	4	
W_1_	T	*An. arabiensis*	2	1	3	1	1.75 ± 0.47
		*Ae. aegypti*	0	0	0	1	0.25 ± 0.25
	C	*An. arabiensis*	33	41	29	36	34.75 ± 2.52
		*Ae. aegypti*	10	11	13	17	12.75 ± 1.54
W_2_	T	*An. arabiensis*	1	2	1	3	1.75 ± 0.47
		*Ae. aegypti*	1	0	1	1	0.75 ± 0.25
	C	*An. arabiensis*	22	39	29	25	28.75 ± 3.70
		*Ae. aegypti*	4	9	1	7	5.25 ± 1.75
W_3_	T	*An. arabiensis*	2	1	4	2	2.25 ± 0.62
		*Ae. aegypti*	0	1	2	0	0.75 ± 0.47
	C	*An. arabiensis*	39	20	34	40	33.25 ± 4.60
		*Ae. aegypti*	2	1	3	4	2.5 ± 0.64
W_4_	T	*An. arabiensis*	4	2	1	0	1.75 ± 0.85
		*Ae. aegypti*	1	0	0	0	0.25 ± 0.25
	C	*An. arabiensis*	22	36	40	35	33.25 ± 3.90
		*Ae. aegypti*	1	1	3	2	1.75 ± 0.47

SE: Standard error. T: Treatment. C: Control. W: Week.

**Table 4:** The mean repellent index (R) of mosquitoes collected at human bait in control and treatment huts by thermal expulsion.

The mosquito species	Conditions	Mean number collected	The mean repellent index (R)	P-Value∗
*An. arabiensis*	Treatment	7.5	94.23	P = 0.0001
	Control	130		
*Ae. aegypti*	Treatment	2	91	P = 0.0012
	Control	22.25		

∗P**-**Value obtained from Tukey's test at α = 0.05.

The mean number of mosquitos collected, in both treatment, as well as control huts, and the mean smoke repellency index (R) of *Ae. aegypti* and *An. arabiensis* by direct burning are shown in Table 1. The mean repellency index (R) of the smoke against *An. arabiensis* was 93.27% and against *Ae. aegypti was* 93.26%. Repellency between treatment and control groups were reduced significantly (P < 0.05).

The mean number of mosquitos collected in both treatment and control huts and the mean smoke repellent index (R) against *Ae. aegypti* & *An. arabiensis* by thermal expulsion in all treatment nights are shown at Table 3. The mean repellent index (R) of the smoke against *An.* *arabiensis* and *Ae. aegypti* was 94.23% and 91, respectively (P < 0.05).

A total of 1430 mosquitoes were collected during the treatment nights, of which *An. arabiensis* constituted the bulk of the collection (79.51%), *Ae. aegypti* (14.54%), *An*. *pharoensis* (3.5%) and *An*. *funestus* (2.45%). *An*. *pharoensis* and *An*. *funestus* were not included in analysis due to low sample sizes. The smoke from plants powder was significantly reduced the number of mosquito landings (P < 0.05) in both methods of application compared to the control.

Specific investigations were not conducted to test the individual repelling effect of the mixed power of the Neem (*Azadirachta indica*), Redgum (*Eucalyptus camaldulensis*) and Basil (*Ocimum forskolei*) by direct burning and thermal explusion, although both methods of application provided important and significantly higher degree of repellence in comparison with other plant species reported.

## 4 Discussion

There are a number of preventive measures that have been used by different communities to control mosquitoes. In Ethiopia, spraying pesticides (primarily DTT), biological control, long-lastinginsecticide-treated mosquito nets (LLITNs) and environmental management efforts are the main mosquitoes controlling measures (Gari and Lindtjørn, 2018; Asale et al., 2019).

The use of plants with repellent property to reduce human vector contact is common practice in village communities. Smoke is the most widely used means for reducing mosquito bites. The majority of plants used as repellents are *Ocimum* species (*Ocimum forskolei, Ocimum kilimandscharicum* and *Ocimum* *suave*), *Eucalyptus* species, (*Eucalyptus camaldulensis*), *Lantana camara* and *Azadirachta* *indica*.

Plants may be alternative source for mosquito repellent agents since they constitute a rich source of bioactive chemicals (Wink, 1993). Plant products can either be used as an insecticide or as repellents against mosquito bites; depending on the type of activity they demonstrate (Mittal and Subbarao 2003). Lemon eucalyptus (*Corymbia citriodora*), *Eucalyptus camaldulensis*, *Ocimum suave* and *Ocimum basilicum* have been evaluated for their repellency by thermal expulsion of their leaves from traditional stoves against *An. arabiensis* and *An. pharoensis* in central part of Ethiopia (Dugassa et al., 2009). In their report, *Ocimum basilicum* was found to be the most effective repellent plant against *An. arabiensis* by the direct burning application method (73.11%) followed by *Ocimum suave* (71.51%), *Corymbia citriodora* (70.59%) and *Eucalyptus camaldulensis* (65.29%). In the thermal expulsion method, similar results were obtained as *Corymbia citriodora* (78.69%) and *Ocimum* basilicum (78.66%) showed similar repellency effects followed by *Ocimum suave* (73.55%) and *Eucalyptus camaldulensis* (71.91%) against *An. arabiensis*. All plant species tested showed over 72% repellency by the thermal expulsion application method against *An. pharoensis* (Dugassa et al., 2009). Similar study was conducted in traditional village houses in western Kenya to evaluate the repellency of Lemon eucalyptus (*Corymbia citriodora*) and other repellent plants (*Ocimum suave* and *O. Itilimands charicum*) against *An. gambiae* and *An. funestus* by thermal expulsion of their leaves from traditional stoves (Seyoum et al., 2003). The highest repellency of was 49% by *Corymbia citriodora* against *An. gambiae* and only 15% repellency against *An. funestus*.

Leaves of *Corymbia* *citriodora* exhibited the highest repellency (51.3%) by direct burning, followed by leaves of *Lantana uckambensis* (33.4%) and, leaves and seeds of *Ocimum suave* 28.0%. However, combination of *Ocimum kilimandscharicum* with *Lantana uckambensis* increased repellent effect to 54.8% by thermal expulsion (Seyoum et al., 2002a). Seyoum et al. (2002b) performed similar experiments in semi-field environment in western Kenya against *An. gambiae*. They observed the highest repellency of 74.5% and 51.3% for *Corymbia citriodora* by thermal expulsion and direct burning, respectively. Our results by thermal expulsion and direct burning in field situations against both *An. arabiensis* and *Ae. aegypti* in the study area was similar to results by Seyoum et al. (2002a,b). *Ocimum forskolei* reduced biting by over 50% against *An. arabiensis* under field conditions according to Waka et al. (2004). *Ocimum suave* and *Ocimum* *kilimandscharium* are used extensively in Tanzania, and are highly effective in bioassays against a range of mosquito species (Kweka et al., 2008). In addition, there are reports of *Ocimum* spp. being used as mosquito repellents via burning or thermal expulsion (Tawatsin et al., 2001).

There are well established variations in the susceptibility of mosquito species to synthetic repellents like DEET (Tawatsin et al., 2001). DEET is a synthetic mosquito repellent widely used all over the world for protection against biting mosquito. Because of concerns of the side effects of DEET, the U.S. Centers for Disease Control and Prevention (CDC) licensed plant-based extracts as mosquito repellents, one of which was PMD (Para-Methane-3,8 diol) (Carroll and Loye, 2006). In developed nations, PMD was successfully commercialized and is widely used (Curtis et al., 1990). Several plants and plant varieties are known to produce a range of oils that have been shown to be effective mosquito repellents. People burn orange peels in Sierra Leone and Ghana to repel mosquitoes, while neem leaves (*Azadirachta indica*) and baobab tree leaves (*Adansonia digitata*) are burned in Ghana and Gambia (Aikins et al., 1993). The Neem tree (*Azadirachta indica*) products were well-known for insect repellent and antifeedant properties long before the advent of synthetic insecticides and have already been documented using its various components in agriculture and other areas (Rutledge et al., 1983; Schreck, 1995; Bhoopendra et al., 2013). The burning of fresh and dried leaves from Lamiaceae, Poaceae and Pinaceae around and within the home to provide protection against mosquito bites is widely used throughout rural Ethiopia (Karunamoorthi et al., 2009a,b). Some essential chemical components like eugenol, linalool, and methyl cinnamate have also been reported. These plants usually contain camphor and thymol that demonstrate mosquito repellent properties (Craveiro et al., 1981). Essential oil from this plant repelled *Ae. aegypti* for about 75 min (Tawatsin et al., 2001) and was also reported to have insecticidal activity against a variety of insects (Shaaya et al., 1997). It was also shown that *Ocimum* *forsltolei* reduced indoor biting activity against *An. arabiensis* by 53% when its fresh leaves and shoots were hanging at the ends of the beds in Eritrea (Waka et al., 2004).

The higher percentage of repellency (>90%) was generally observed in the present study might have some contributions from the burning charcoal itself used to smolder the plants. Burning charcoal may provide some degree of protection (approximately 20%) from mosquitoes, probably by reducing humidity near the fire (Moore et al., 2007). Assuming the same level of repellency from charcoal alone in the present study, the actual repellencies of the test plants could be greater than 70% by both methods of applications which still are comparative with work of Seyoum et al., 2002a, Seyoum et al., 2002b, 2003) and Dugassa et al. (2009). However, additional repellents provided by burning charcoal, firewood, or dried cow dung are usually used in traditional stoves in Ethiopia to heat the desired plant parts or incense (Dugassa et al., 2009).

Plants that demonstrate repellent activity have an important place in protecting man from the bites of insect pests. Effective repellents are useful in reducing man-vector contact and in the interruption of transmission of phatogens. Mosquito repellents may be one of the most effective tools for protecting human from vector-borne diseases and nuisance biting mosquitoes (Curtis et al., 1990). Repellent compounds should be non toxic, non irritating and long lasting (Kalyana-sundaram and Babu, 1982). Repellents are substances that act as area repellents (at a distance) by deterring insects from flying to, landing on or biting human, or are applied to exposed skin and clothing for protection (Brown and Hebert, 1997; Blackwell et al., 2003; Choochote et al., 2007). Many natural products are safe for humans when compared to synthetic chemical pesticides (Sharma and Ansari, 1994; Regnault-Roger et al., 2012; Wendimu and Tekalign, 2020). Property utilized repellents are an inexpensive means of reducing or preventing arthropod-borne diseases and nuisance biters on a wide range of vectors (Gupta and Rutledge, 1994).

### 4.1 Health hazards due to the repellents

There has been much debate about using plants and plant extracts as repellents against mosquitoes and other biting arthropods. However, one primary factor is "safety". Just because they/it has been used for millennia, doesn't mean that it is safe to use although it may be better than the alternative of getting malaria or some other arthropod-borne disease. To study ill effects of the repellents on human health, of the total eight volunteers interviewed report not any complaint of adverse health impact in the use of repellents, however, they pointed out, eye irritation and sneezing were sometimes encountered with moderate breathing problems.

### 4.2 Completely safe alternative measures to combat mosquitos

There are a number of completely safe alternate measures apart from using plants and plant extracts and other chemical-based repellents to control mosquitoes. Use of these requires personal attention and involvement of the community and the local bodies. They are (i) Source reduction: the regular emptying of nearby water sources around the huts and drying wells that can be used as mosquito breeding grounds. (ii) Drainage: preparing drainage tubes or ditches to eliminate standing water in drains away from the living environment. (iii) Personal prevention methods: long-lasting insecticide-treated mosquito nets (LLITNs) may be best used indoor. However, not more than 50 g of Neem (*Azadirachta* *indica),* Redgum (*Eucalyptus camaldulensis)* and Basil (*Ocimum forskolei*) plant leaves powder mixture can be used for 40–50 min as the above protective measures cannot be taken in resource-poor rural communities.

## 5 Conclusion

The repellent effects of selected plants viz. Neem (*Azadirachta indica*), Red gum (*Eucalyptus camaldulensis*) and Basil (*Ocimum forskolei*) leave powder significantly reduced biting activity of two species of mosquitoes. Repellents from herbal combination pose significant effect to repelled mosquitoes. Moreover, the repellents are safe, eco-friendly, cheap, easy to use and has maximum area repellency against mosquitoes. The data in relation to acute and chronic toxicity of the selected medicinal plants has not been determined. In addition, home-made herbal repellents maybe less harmful to health than insecticide coils and other synthetic chemicals available in the market. Leaf products prepared at home because they do not require heavy infrastructure and investment as compared to coils and mats containing synthetic products. The community-wide use of such repellent plants has potential to complement existing control measures, such as ITNs (Insecticide treated mosquito nets). The two methods of application may offer cost-efficient alternatives as an additional household protection and as a helpful addition to bed nets, especially for the early part of the evening before bedtime.

### 5.1 Limitations

In this particular study the smoke treated huts were assigned as an experiment groups (T) and the huts with no particular treatment were assigned as a control huts (C). But for this study we haven't used smoking from any plants to check the result of smoking from the target plants (T) as if we get the same result if we used any plant in the area.

## Declarations

### Author contribution statement

Abenezer Wendimu: Conceived and designed the experiments; Performed the experiments; Analyzed and interpreted the data; Contributed reagents, materials, analysis tools or data; Wrote the paper.

Wondimagegnehu Tekalign: Conceived and designed the experiments; Contributed reagents, materials, analysis tools or data; Wrote the paper.

### Funding statement

This research did not receive any specific grant from funding agencies in the public, commercial, or not-for-profit sectors.

### Data availability statement

Data will be made available on request.

### Declaration of interests statement

The authors declare no conflict of interest.

### Additional information

No additional information is available for this paper.
